# Variant detection and runs of homozygosity in next generation sequencing data elucidate the genetic background of Lundehund syndrome

**DOI:** 10.1186/s12864-016-2844-6

**Published:** 2016-08-02

**Authors:** Julia Metzger, Sophia Pfahler, Ottmar Distl

**Affiliations:** Institute for Animal Breeding and Genetics, University of Veterinary Medicine Hannover Foundation, Hanover, Germany

**Keywords:** *LEPREL1*, Lundehund syndrome, Runs of homozygosity

## Abstract

**Background:**

The Lundehund is a highly specialized breed characterized by a unique flexibility of the joints and polydactyly in all four limbs. The extremely small population size and high inbreeding has promoted a high frequency of diseased dogs affected by the Lundehund syndrome (LS), a severe gastro-enteropathic disease.

**Results:**

Comprehensive analysis of bead chip and whole-genome sequencing data for LS in the Lundehund resulted in a genome-wide association signal on CFA 34 and LS-specific runs of homozygosity (ROH) in this region. Filtering analysis for variants with predicted high or moderate effects revealed a missense mutation in *LEPREL1* 1.2 Mb proximal to the region of the genome-wide association, which was shown to be significantly associated with LS. LS-affected Lundehund harbored the mutant *LEPREL1*:g.139212C>G genotype A/A whereas all controls of other breeds showed the C/C wild type.

In addition, ROH analysis for the Lundehund indicated a high enrichment of genes in potential signatures of selection affecting protein activation and immunoregulatory processes like *NOD1* potentially involved in LS breed disposition.

**Conclusions:**

Sequencing results for Lundehund specific traits reveal a potential causative mutation for LS in the neuropeptide operating gene *LEPREL1* and suggests it as a precursor of the inflammatory process. Analyses of ROH regions give an insight into the genetic background of characteristic traits in the Lundehund that remain to be elucidated in the future.

**Electronic supplementary material:**

The online version of this article (doi:10.1186/s12864-016-2844-6) contains supplementary material, which is available to authorized users.

## Background

The Norwegian Lundehund represents a specific but small group in the variety of dog breeds which have evolved since domestication [1]. It stands out by exceptional characteristics like double dewclaws and extreme flexibility in shoulder and neck, which represent traits fixed in the Lundehund population [2]. In addition, the Lundehund harbors a breed disposition for a syndrome comprising particular features of protein-losing enteropathy (PLE), intestinal lymphangiectasia, gastrointestinal disturbance, inflammatory bowel disease and malabsorption designated as Lundehund syndrome (LS) [3, 4]. Clinical signs are diarrhea, vomiting, weight loss, edema and apathy often accompanied with decreased concentrations of albumin and globulin in blood profile [2]. A similar condition has been described in the Soft Coated Wheaten Terrier affecting immune system, gut and kidney [5, 6]. It was supposed that one or more genes involved in these complex systems might be responsible for a breed disposition for PLE. An increased occurrence of PLE could also be found in Rottweilers and Yorkshire terriers [7–9]. In Basenji and German Shepherd, hypoalbuminemia and hypoglobulinemia was found in dogs with severe lymphocytic-plasmacytic enteritis characterized by excessive infiltrates of mononuclear inflammatory cells [10, 11]. It was suggested that the inflammatory bowel disease in German Shepherd was the result of a complex etiology with the involvement of different variant effects similar to Crohn’s disease, the chronic inflammatory bowel disease in human [12–15].

In the Lundehund, a signature of selection for LS was suggested to be located on CFA 9 in the region of *Caspase Recruitment Domain Family, Member 9* (*CARD9),* which is known to be associated with Crohn’s disease and ulcerative colitis in human [16, 17]. In addition, extended haplotype homozygosity (EHH) tests suggested further breed specific characteristics like polydactyly, body size or flexibility of the joints as targets of specific selection [17]. Genotyping the *limb development membrane protein 1 (LMBR1)* mutation DC-2, which was shown to be associated with preaxial polydactyly in western dogs, revealed the mutant genotype (A/A) in all tested Lundehund [18, 19]. In general, the frequency of long stretches of homozygous genotypes on basis of bead chip data was shown to be high which was suggested to be a result of an extremely low genetic variability in the Lundehund breed [1, 17, 20].

In this study whole-genome sequencing was performed in two Lundehund pools comprising LS-affected and LS-unaffected individuals. Sequence data were investigated for runs of homozygosity (ROHs) and variants predicted to have high or moderate effects in order to elucidate the genetic background of LS.

## Results

### Phenotype

**Fig. 1:**
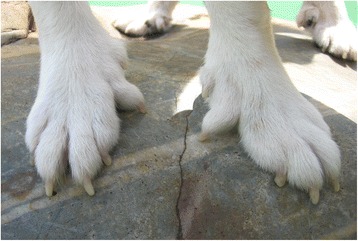
Polydactyly in the Lundehund. Polydactyly with six toes at all four limbs is a characteristic trait in the Lundehund

**Fig. 2:**
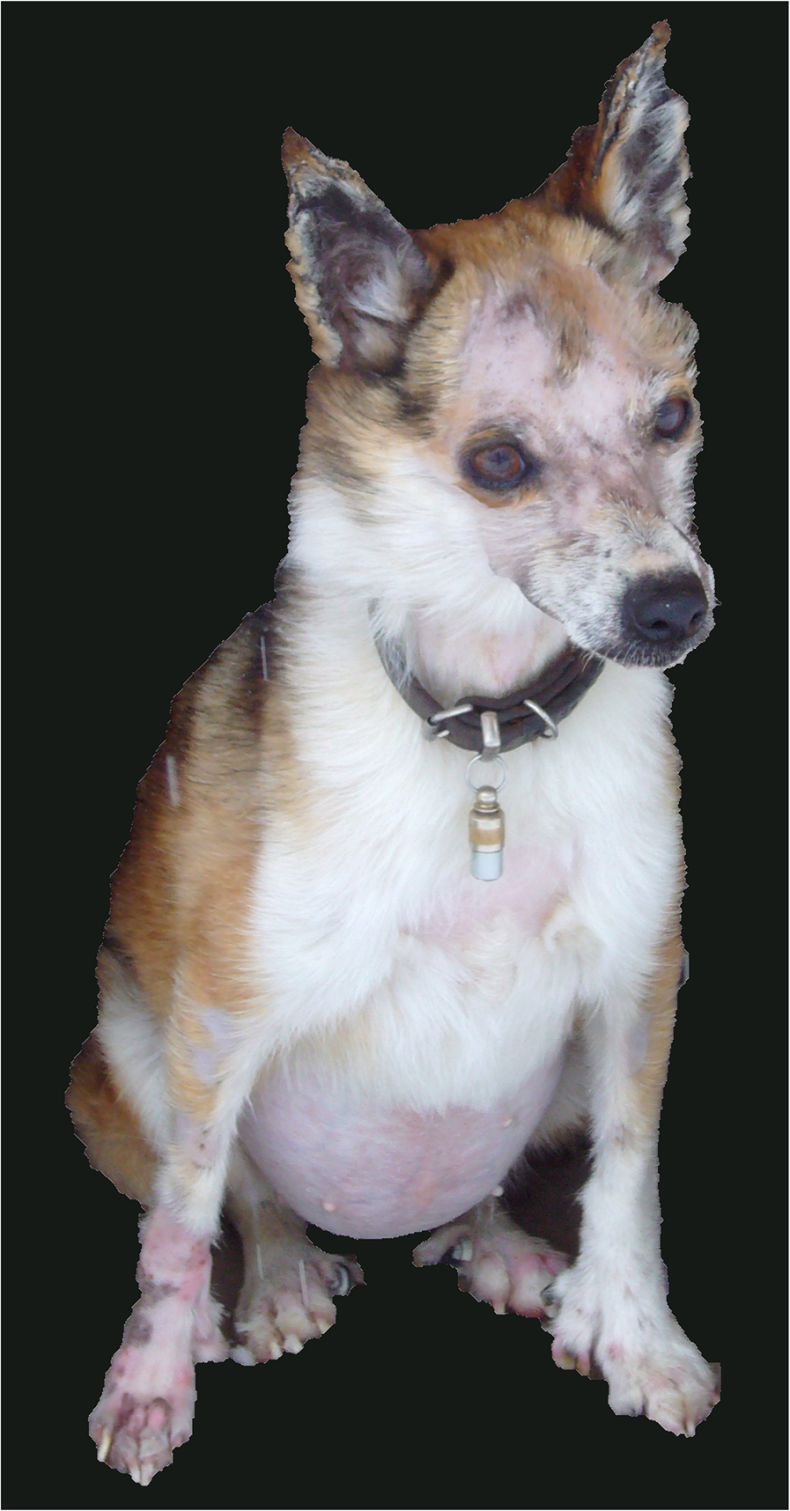
Clinical picture of Lundehund syndrome (LS). A five year old Lundehund with severe signs of LS is shown. Persistent diarrhea, vomiting and ascites resulted in marked weight and hair loss. The dog had to be euthanized due to poor prognosis and unresponsive therapy

Samples of six Lundehund dogs with typical characteristics of a puffin hunter phenotype including an additional toe and joint flexibility (Fig. 1) were chosen for whole-genome sequencing in two pools. One pool enclosed three healthy dogs whereas the other pool comprised three Lundehund showing severe signs of gastroenteropathy known as LS. In addition, 12 LS-affected Lundehund with clinical signs and low blood protein parameters, 6 Lundehund suspected to be LS-affected due to recurrent diarrhea and vomiting and 12 Lundehund without clinical signs of LS classified as LS-unaffected were available for further analysis. Characteristic clinical signs were diarrhea, vomiting, weight loss and apathy (Fig. 2). The onset of first signs varied widely from the age of 2.5 to 10.5 years (Additional file 1). Some cases also showed ascites and edema in the limbs. The results of blood screening revealed hypoalbuminemia in all analyzed cases, sometimes accompanied with hypoglobulinemia, reduced levels of fructosamines and vitamin B, hypo- or hypercalcemia, as well as increased concentrations of serum folate and alanine aminotransferase.

### Pedigree and association analysis

Inspection of the pedigree data of all Lundehund under study revealed close relationships among affected dogs (Additional file 2). In total 24 female and 16 male LS-affected Lundehund were included in this pedigree data. A complex segregation analysis revealed a recessive major gene model as the most likely mode of inheritance with the lowest −2 log-likelihood at 52.68 (Additional file 3).

**Fig. 3:**
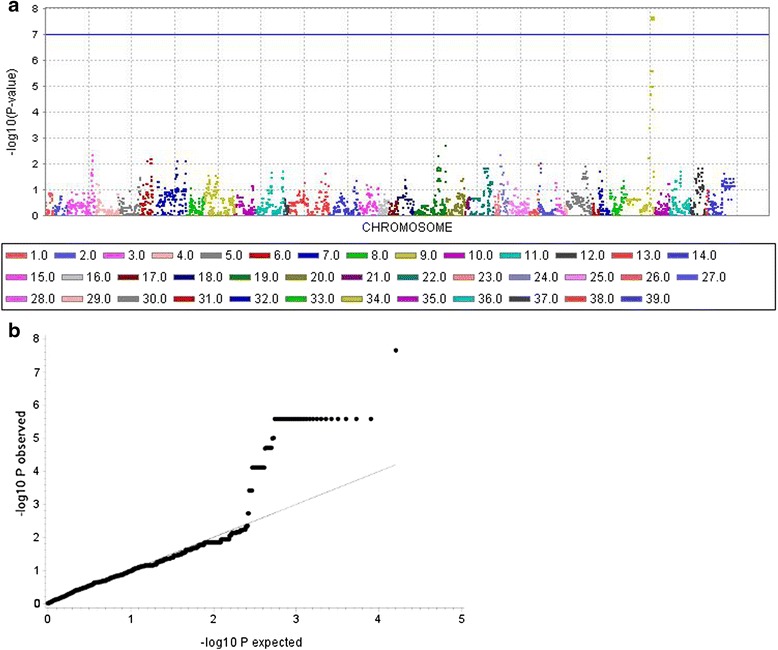
Genome-wide association analysis (GWAS) for Lundehund syndrome (LS). **a** The Manhattan-plot of the -log_10_ *P*-values shows a highly significant peak on canine chromosome (CFA) 34 in the region of 23,373,982-24,488,983 bp (CanFam 3.0). **b** Observed *versus* expected -log_10_ *P*-values (Q-Q plot) reveal 31 SNPs with highly significant -log_10_ *P*-values

Genome-wide association analysis for LS in 17 LS-affected and 8 LS-unaffected Lundehund showed a highly significant peak on CFA 34 at 26,384,304-27,498,705 bp (CanFam 2.0) corresponding to 23,373,982-24,488,983 bp in CanFam 3.1 assembly (Fig. 3) in the region of *fibroblast growth factor 12* (*FGF12*; ENSCAFG00000031187) and *mab-21 domain containing 2* (*MB21D2*; ENSCAFG00000014075). The highest -log_10_P value (−log_10_*P* = 7.7) could be shown for one SNP at 24,152,349 bp whereas further 30 SNPs in the associated region reached -log_10_P-values of 5.6-5.9. Expected *versus* observed -log_10_P-values (quantile-quantile-plot) showed that inflation due to stratification effects had not increased -log_10_P-values. The distribution of genotypes suggested a recessive effect as proposed by segregation analysis.

### Sequencing and variant detection

Whole-genome sequencing of two Lundehund pools with three dogs each resulted in a mean coverage of 15.56X for Lundehund pool 1 (LS-affected) and 14.48X Lundehund pool 2 (LS-unaffected). In total 10,584,482 SNPs and 3,223,567 insertions/deletions (INDEL) could be detected for pool 1 as well as 10,530,165 SNPs and 3,207,163 INDEL for pool 2 in raw data after variant calling. Stringent filters for read depth and quality of each variant led to a set of 8,815,395 SNPs (pool 1) as well as 8,799,793 SNPs (pool 2) for ROH analysis. For these SNPs the mean heterozygosity per site was 0.11 in both pools.

### LS-specific ROH detection

A total of 1713 ROHs specific for LS-affected dogs were detected. Three ROHs could be found directly in the region of association on CFA 34 at 23,223,356-24,490,050 bp harboring the genes *Coiled-Coil Domain Containing 50 (CCDC50)*, *FGF12* and *MB21D2* (Additional file 4). In private ROHs analysis for homozygous regions which could be exclusively found in LS-affected as well as LS-unaffected Lundehund, 472 ROH regions could be detected for pool 1 (LS-affected, Additional file 5) and 408 ROH regions for pool 2 (LS-unaffected, Additional file 6). The largest private ROH region for pool 1 could also be found on CFA 15 in a wider area of 7,812,609-13,628,020 bp including 51 genes.

### Breed specific ROH

Consensus ROH detection was performed to identify potential signatures of selection for unique breed specific characteristics whose intense human selection might be collaterally associated with the high incidence of LS in the Lundehund population.

In total 660 Lundehund specific consensuses ROH regions could be detected for 500-SNP windows (Additional file 7). A notably high number of large stretches of consecutive homozygous genotypes was found on CFA 12 in the region of 32,968,627-49,633,816 bp and CFA 7 in the region of 28,049,903-54,984,739 bp. These regions harbored a large number of genes including *PRRX1 (paired related homeobox 1)* and *GREM2 (gremlin 2, DAN family BMP antagonist)* on CFA 7. Furthermore, we detected a ROH region on CFA 14 at 42,891,687-43,618,177 bp harboring *Nucleotide-Binding Oligomerization Domain Containing 1 (NOD1)*. In contrast, no ROH region could be detected harboring the candidate gene *LMBR1 (limb development membrane protein 1)* for canine polydactyl mutations [19]. Further genotyping of the intronic *LMBR1* SNP DC-2 revealed the mutant DC-2 allele homozygous in all analyzed Lundehund but also in the Norwegian Buhund, Bernese Mountain Dogs, Briard and Irish Wolfhound (Additional file 8).

### Variants in ROH regions

Detection of SNPs or INDEL predicted to have high or moderate effects in ROH regions was performed to identify LS-specific or Lundehund-specific variants. No variant with predicted high or moderate effects could be detected within *FGF12* and *MB21D2* located in the region of ROHs found in the LS-affected (pool 1) which matched the region of genome-wide association. In total 2036 intron variants with a homozygous mutant genotype in LS-affected Lundehund, one 3′UTR and 13 5′UTR variants could be found in *FGF12* with predicted low or modifier effects only. In *MB21D2* no LS-associated variant could be detected.

Analysis of private LS-specific ROH regions resulted in one variant with a homozygous mutant genotype in LS-affected Lundehund, a heterozygous genotype in LS-unaffected Lundehund and a wildtype genotype in controls. This missense variant *COL28A1*:g.159951T>A on ECA14 was predicted to be deleterious (SIFT) as well as probably damaging (PolyPhen).

In ROH regions detected in all Lundehund (pool 1 and 2) seven variants with predicted tolerated or deleterious (low confidence) effects could be found. The candidate genes *PRRX1* and *GREM2* did not harbor variants with predicted high or moderate effects. Nevertheless, ten intronic variants could be detected in *PRRX1* with mutant homozygous genotypes exclusively found in the Lundehund pools.

### Functional annotation of ROH regions

Functional gene classification of human orthologues detected in ROH regions showed a particularly high involvement in biological processes affecting cellular processes (GO:0009987), localization (GO:0051179), response to stimulus (GO:0050896) and metabolic processes (GO:0008152) in all data sets (Additional file 9). PANTHER overrepresentation test in ROH regions in LS-affected Lundehund revealed an enrichment of genes involved in cellular process (GO:0009987, GO:0006464), metabolic process (GO:0008152, GO:0019538, GO:0044238, GO:0019219) and transcription (GO:0006351). Private ROHs detected in LS-affected dogs revealed a >5 fold enrichment of genes known to play a role in negative regulation of complement activation (GO:0045916), protein activation cascade (GO:2000258), humoral immune response (GO:0002921) and also in the perception of taste (GO:0001580, GO:0050913, GO:0050912, GO:0050909). In consensus Lundehund ROH regions genes affecting single-organism (GO:0044763) as well as cellular (GO:0009987) and biological (GO:0008150) processes were predicted to be overrepresented (Table 1).

**Table 1:** Statistical overrepresentation test for biological processes. All human orthologues derived from ROH detection were analyzed for an enrichment of genes involved in specific processes using PANTHER tools. The number of detected genes in ROH regions in 500-SNP windows and Gene Ontology (GO) terms are shown for Bonferroni corrected significant P-values <0.05

	Number of reference genes (Homo sapiens)	ROH regions in LS-affected: expected	Bonferroni corrected P-value for multiple testing	Private ROH regions in LS-affected: expected	Bonferroni corrected P-value for multiple testing	Private ROH regions in LS-unaffected: expected	Bonferroni corrected *P*-value for multiple testing	Lundehund consensus ROH regions: expected	Bonferroni corrected P-value for multiple testing
Single-organism cellular process (GO:0044763)	11415	─	─	─	─	─	─	480.42	1.03E-06
Single-organism process (GO:0044699)	12755	─	─	─	─	─	─	536.82	5.36E-06
Cellular process (GO:0009987)	14147	1714.55	3.37E-05	─	─	─	─	595.41	4.70E-07
Biological process (GO:0008150)	16542	─	─	─	─	─	─	696.2	1.02E-04
Generation of neurons (GO:0048699)	1551	─	─	─	─	61.18	1.61E-02	─	─
Neurogenesis (GO:0022008)	1628	─	─	─	─	64.22	2.46E-02	─	─
Cellular component organization or biogenesis (GO:0071840)	5188	─	─	─	─	204.64	1.47E-02	─	─
Cellular protein modification process (GO:0006464)	1317	336.62	1.15E-03	─	─	─	─	─	─
Protein metabolic process (GO:0019538)	2692	688.07	2.95E-04	─	─	─	─	─	─
Metabolic process (GO:0008152)	8247	2107.91	7.09E-04	─	─	─	─	─	─
Primary metabolic process (GO:0044238)	6825	1744.45	1.39E-02	─	─	─	─	─	─
Transcription, DNA-dependent (GO:0006351)	1941	496.11	4.71E-03	─	─	─	─	─	─
Regulation of nucleobase-containing compound metabolic process (GO:0019219)	1700	434.52	1.03E-02	─	─	─	─	─	─

### Filtering for mutations with predicted high or moderate effects

Specific filtering for variants with predicted high or moderate effects in the region of genome-wide association for LS revealed no variant directly in this region but one missense mutation 1.2 Mb proximal to the peak of association in the candidate gene *LEPREL1* (Table 2). It was predicted to result in a substitution glutamic acid to glutamine.

**Table 2:** Filtered variants from whole-genome analysis. Variants with predicted high or moderate effects and a homozygous mutant genotype exclusively found in the LS-affected pool as well as a heterozygous or homozygous wild-type genotype in the LS-unaffected pool are shown. All five reference dogs of four different breeds do not show the mutant genotype. Potential functional effects of these variants were predicted using SIFT and PolyPhen

CFA	Position	Base change	Amino acid change	Consequence	Genotype (LS-affected Lunde-hund pool)	Genotype (LS-unaffected Lunde-hund pool)	Genotype (5 reference dogs)	Gene (transcript)	SIFT	PolyPhen-2
1	13398018	T>G	T>P/ S>R	missense variant	1/1	0/0	0/0	ENSCAFG00000031329 (novel gene; (ENSCAFT00000043665) and ENSCAFG00000030129 (novel gene; ENSCAFT00000048679)	tolerated (0.07)/ deleterious (0.02)	benign (0.074)/-
1	111903572	A>G	T>A	missense variant	1/1	0/1	0/0	CEACAM1 (ENSCAFT00000007749/ ENSCAFT00000046087/ ENSCAFT00000022623/ ENSCAFT00000047331/ ENSCAFT00000049292)	tolerated (0.08/ 0.13/ 0.2/ 0.21/ 0.2)	benign (0.00)/ benign (0.310)/ possibly damaging (0.659)/ possibly damaging (0.605)/ benign (0.250)
3	56483857	G>T	P>T	missense variant	1/1	0/1	0/0	IL16 (ENSCAFT00000021964)	tolerated (0.59)	benign (0.310)
3	62265062	A>T	F>I	missense variant	1/1	0/1	0/0	ENSCAFG00000017475 (novel gene; ENSCAFT00000027691)	deleterious (0.01)	possibly damaging (0.614)
5	16233038	G>T	L>M	missense variant	1/1	0/1	0/0	CEP164 (ENSCAFT00000020686)	deleterious (0.02)	probably damaging (0.996)
6	67204999	C>T	R>Q	non coding transcript variant	1/1	0/1	0/0	ENSCAFG00000005648 (novel gene; ENSCAFT00000009086)	-	-
6	67205308	A>C	Y>* (stop codon)	non coding transcript variant	1/1	0/1	0/0	ENSCAFG00000005648 (novel gene; ENSCAFT00000009086)	-	-
6	67205754	G>A	R>* (stop codon)	non coding transcript variant	1/1	0/1	0/0	ENSCAFG00000005648 (novel gene; ENSCAFT00000009086)	-	-
6	67205959	A>G	L>P	non coding transcript variant	1/1	0/1	0/0	ENSCAFG00000005648 (novel gene; ENSCAFT00000009086)	-	-
6	67205966	T>G	T>P	non coding transcript variant	1/1	0/1	0/0	ENSCAFG00000005648 (novel gene; ENSCAFT00000009086)	-	-
7	2590167	G>A	S>N	missense variant	1/1	0/1	0/0	KIF14 (ENSCAFT00000017720)	tolerated (0.21)	benign (0.002)
7	4169250	A>T	F>Y	missense variant	1/1	0/1	0/0	PTPRC (ENSCAFT00000017964/ ENSCAFT00000017955)	tolerated (0.3/ 0.31)	benign (0.023/ 0.347 )
8	22616300	C>T	P>L	missense variant	1/1	0/1	0/0	FANCM (ENSCAFT00000046644/ ENSCAFT00000022327/ ENSCAFT00000048988)	tolerated (0.42/ 0.34/ 0.42)	benign (0.009/ 0.009/ 0.004 )
8	47544408	G>A	G>R	missense variant	1/1	0/1	0/0	VRTN (ENSCAFT00000026784)	tolerated (0.56)	possibly damaging (0.560)
8	73685892	C>G	Q>H	missense variant	1/1	0/1	0/0	ENSCAFG00000029996 (novel gene; ENSCAFT00000049952)	deleterious (0)	benign (0.152)
8	73685923	C>G	W>S	missense variant	1/1	0/1	0/0	ENSCAFG00000029996 (novel gene; ENSCAFT00000049952)	deleterious (0)	probably damaging (1.000)
8	73685927	T>G	S>R	missense variant	1/1	0/1	0/0	ENSCAFG00000029996 (novel gene; ENSCAFT00000049952)	tolerated (0.21)	probably damaging (0.963)
9	37739213	C>T	R>W	missense variant	1/1	0/1	0/0	ENSCAFG00000032731 (novel gene; ENSCAFT00000047013)	tolerated (0.07)	probably damaging (0.998)
9	51227945	T>C	H>R	missense variant	1/1	0/1	0/0	ENSCAFG00000019863 (novel gene; ENSCAFT00000031603)	deleterious (0)	possibly damaging (0.898)
10	36330685	C>T	A>V	missense variant	1/1	0/1	0/0	FAM32A (ENSCAFT00000003279)	tolerated (0.16)	benign (0.328)
11	67454670	A>C	S>R	missense variant	1/1	0/1	0/0	ENSCAFG00000028946 (novel gene; ENSCAFT00000045538)	tolerated (0.33)	-
12	40430108-40430109	A>AGG	R47fs	frameshift variant	1/1	0/1	0/0	ENSCAFG00000030790 (novel gene; ENSCAFT00000045206)	-	-
12	52354031	A>G	F>S	missense variant	1/1	0/1	0/0	ENSCAFG00000030881 (novel gene; ENSCAFT00000046435)	deleterious (0.01)	-
12	52354041	G>A	L>F	missense variant	1/1	0/1	0/0	ENSCAFG00000030881 (novel gene; ENSCAFT00000046435)	deleterious (0.02)	-
14	16187782	T>G	I>M	missense variant	1/1	0/1	0/0	CFAP69 (ENSCAFT00000002973)	tolerated (0.23)	possibly damaging (0.648)
14	22993351	A>T	C>S	missense variant	1/1	0/1	0/0	COL28A1 (ENSCAFT00000044570)	deleterious (0)	probably damaging (1.000)
18	39577830	C>T	A>V	missense variant	1/1	0/1	0/0	ENSCAFG00000030998 (novel gene; ENSCAFT00000047197)	tolerated (1)	benign (0.000)
20	54722514 rs22884799	T>C	I>M	missense variant	1/1	0/1	0/0	KDM4B (ENSCAFT00000030040)	tolerated (0.7)	possibly damaging (0.728)
27	2322565	C>T	R>C	missense variant	1/1	0/1	0/0	KRT3 (ENSCAFT00000011634)	deleterious (0.03)	possibly damaging (0.942)
28	31706107	A>C	N>T	missense variant	1/1	0/1	0/0	ENSCAFG00000012412 (novel gene; (ENSCAFT00000019700)	tolerated (0.78)	benign (0.000)
32	21555092	C>T	D>N	missense variant	1/1	0/1	0/0	TRMT10A (ENSCAFT00000016732)	tolerated (0.31)	probably damaging (0.997)
34	22046092	C>G	E>Q	missense variant	1/1	0/1	0/0	LEPREL1 (ENSCAFT00000022188)	tolerated (0.43)	possibly damaging (0.945)
X	96702059 rs24643372	A>G	R>G	missense variant	1/1	0/1	0/0	ENSCAFG00000029138 (novel gene; ENSCAFT00000044717)	tolerated (0.41)	benign (0.001)

* is the official HGVS-symbol for a stop codon

In addition, specific filtering was done for further candidate variants which could potentially be involved in disease development of LS and might have been missed in the specified ROH detection windows or association analysis. We extracted genetic variants putatively associated with LS with predicted high or moderate effects which showed a homozygous mutant genotype only in LS-affected and a heterozygous mutant or wildtype genotype in LS-unaffected dogs. In total 32 SNPs and one INDEL resulted from filtering analysis.

Based on these results we chose the missense mutation *LEPREL1*:g.139212C>G near the region of genome-wide association and further six single nucleotide variants (SNV) in other genomic regions which were predicted to be deleterious (SIFT [21]) as well as possibly or probably damaging (PolyPhen [22]) for validation in all Lundehund samples and across several dog breeds. Three of these SNVs located in regions of an extreme high density of mutations, probably a result of inaccurate gene annotation, were assumed to be false positive results and therefore omitted from analysis. Genotyping of the remaining four non-synonymous SNVs in the region of the candidate genes *leprecan-like 1 (LEPREL1), centrosomal protein 164 kDa (CEP164), collagen, type XXVII, alpha 1 (COL28A1) and keratin 3 (KRT3)* in 36 Lundehund revealed a significant association of *CEP164*:g.57380G>T and *LEPREL1*:g.139212C>G with LS (Table 3).

**Table 3:** Genotypic distribution of candidate SNPs for LS in Lundehund dogs. Chi-square test results are shown for the four candidate SNPs chosen from filtering analysis for mutations predicted to have high or moderate effects. Genotypes are assigned to Lundehund without signs of LS (0), LS-affected Lundehund (1) and LS-suspicious dogs (1 susp.) due to clinical signs

CFA	Gene	Polymorphism	Chi-Square genotype (Probability)	Chi-Square allele (Probability)	Chi-Square trend (Probability)	LS status affected (1) or unaffected (0)	Genotype 0/0 (number and frequency)	Genotype 0/1 (number and frequency)	Genotype 1/1 (number and frequency)
34	*LEPREL1*	LEPREL1:g.139212C>G	6.807 (*P* = 0.033)	4.959 (*P* = 0.026)	4.293 (*P* = 0.038)	0	1 (2.78)	7 (19.44)	7 (19.44)
						1	0 (0.00)	0 (0.00)	15 (41.70)
						1 (susp.)	1 (2.78)	2 (5.56)	3 (8.34)
5	*CEP164*	CEP164:g.57380G>T	6.264 (*P* = 0.044)	4.538 (*P* = 0.033)	4.412 (*P* = 0.036)	0	4 (11.43)	6 (17.14)	5 (14.29)
						1	0 (0.00)	7 (19.46)	7 (19.46)
						1 (susp.)	0 (0.00)	2 (5.56)	4 (11.12)
14	*COL28A1*	COL28A1:g.159951T>A	0.614 (*P* = 0.736)	0.019 (*P* = 0.889)	0.021 (*P* = 0.883)	0	3 (8.57)	7 (20.00)	5 (14.29)
						1	2 (5.72)	8 (22.80)	4 (11.44)
						1 (susp.)	1 (2.86)	4 (11.44)	1 (2.86)
27	*KRT3*	KRT3:g.2584C>T	3.379 (*P* = 0.184)	2.483 (*P* = 0.115)	2.146 (*P* = 0.143)	0	3 (8.33)	8 (22.22)	4 (11.11)
						1	3 (8.33)	4 (11.12)	8 (22.22)
						1 (susp.)	0 (0.00)	2 (5.56)	4 (11.12)

Further genotyping of 186 dogs of 17 breeds with no signs of gastroenteropathic disease showed only *LEPREL1*:g.139212C>G and *COL28A1*:g.159951T>A to be breed specific mutations for the Lundehund (Additional file 10) and revealed no significant P-value for *COL28A1*:g.159951T>A, but a significant P-value of 1.503E-29 for *LEPREL1*:g.139212C>G (Table 4). A closer look at the LS-phenotypes showed that all 17 LS-affected Lundhund harbored the homozygous mutant *LEPREL1*:g.139212C>G genotype whereas three of the six dogs which were estimated to be LS-suspicious due to clinical signs, did not show the homozygous mutant genotype.

**Table 4:** Chi-square test for Lundehund dogs and seventeen different breeds. Other breeds are used as controls for Lundehund-specific LS. The test results are shown for the four candidate SNPs chosen from filtering analysis for mutations predicted to have high or moderate effects

CFA	Gene	Polymorphism	Chi-Square genotype (Probability)	Chi-Square allele (Probability)	Chi-Square trend (Probability)
34	*LEPREL1*	LEPREL1:g.139212C>G	132.735 (*P* = 1.503E-29)	238.686 (*P* = 7.607E-54)	130.861 (*P* = 2.655E-30)
5	*CEP164*	CEP164:g.57380G>T	83.884 (*P* = 6.092E-19)	120.165 (*P* = 5.822E-28)	83.716 (*P* = 5.712E-20)
14	*COL28A1*	COL28A1:g.159951T>A	101.034 (*P* = 1.150E-22)	116.566 (*P* = 3.573E-27)	79.531 (*P* = 4.748E-19)
27	*KRT3*	KRT3:g.2584C>T	3.379 (*P* = 0.184)	2.483 (*P* = 0.115)	2.146 (*P* = 0.173)

### Candidate gene sequencing

Sequence analysis of *LEPREL1* cDNA confirmed the missense mutation in exon 13 in LS-affected Lundehund dogs. No further SNVs could be found in *LEPREL1* to be associated with LS (Additional file 11). Sequencing the complementary DNA (cDNA) of *MB21D2* in an affected Lundehund and a reference German Shepherd dog revealed three mutations. Nevertheless, none of these mutations could be exclusively found in the Lundehund. The predicted gene *FGF12* cDNA could not be amplified at all.

## Discussion

Whole-genome sequencing analysis in two pools of Lundehund dogs revealed a candidate gene for LS-disposition and gave evidence for potential LS-related signatures of selection. The genome-wide association analysis resulted in a significant peak on CFA 34 which could also be shown to be located in a ROH region for LS-affected dogs. Screening of whole-genome data did not reveal any LS-associated mutations with predicted high impacts on protein function but the missense mutation *LEPREL1*:g.139212C>G 1.2 Mb proximal of the region of significant genome-wide association. We assume that the shift of the peak of LS-association and LS-associated ROH region might be a result of the low bead chip marker density that could be found in the region of *LEPREL1.* Furthermore, it could be shown that the detection of ROHs is strongly dependent on the window size and marker density [23] so that not all homozygous stretches can be detected in 500-SNP windows. We assume that smaller windows could probably have enabled the detection of a ROH region comprising *LEPREL1* but might have increased the number of false positive results, too. According to the suggested recessive mode of inheritance the missense mutation *LEPREL1*:g.139212C>G could be shown to be homozygous in all analyzed dogs in NGS data. LS-affected dogs showed the mutant genotype G/G whereas LS-unaffected controls of other breeds did harbor the homozygous wild type C/C. In addition, seven Lundehund with no clinical signs for LS also showed the mutant genotype G/G. Due to the variable onset of the disease it can be proposed that these dogs will develop signs of the disease later in their life. We suggest this SNV as a potential causative mutation for a genetic disposition for LS.

*LEPREL1* was shown to be expressed in a subpopulation of neuroendocrine cells of the intestinal mucosa and suggested to be important for processing and secretion of neuropeptides [24]. It was proposed that the enteric nervous system could trigger the occurrence of inflammatory bowel disease through neuropeptide secretion [25]. Neuropeptides were proposed to play a key role in inflammatory bowel diseases [25, 26]. Blockades for these molecules were considered for therapeutic approaches. We assume mutant *LEPREL1* might be an essential precursor for LS in the Lundehund. Lundehund without clinical signs for LS who harbor this mutant RNA could possibly be subclinically affected or fall ill later in their life by specific trigger mechanisms. It was shown that not a few Lundehund who appeared healthy often had abnormal findings in histopathologic examinations [2]. The onset of the disease could be influenced by external factors triggering phenotypic expression. In the Soft Coated Wheaten Terrier it was proposed that food allergies could play a role in the development of PLE [5, 27]. In addition to external factors, further genes might be involved in triggering LS as well as influence the severity of symptoms. In human Crohn’s disease various mutations have been shown to be associated with disease development in different populations [13, 28–33]. It was suggested that complex regulatory processes driven by various genes could affect the extent of defects and result in different severities.

We assume that further genes might be involved in LS-development and could potentially be related to targets of selection for breed specific traits. This could probably explain the high incidence of LS in the population. In general, ROH analysis for the Lundehund showed a high frequency of long stretches of homozygous genotypes as expected from the extremely low genetic variability of this breed [1]. A high enrichment of genes in these ROH regions affecting protein activation, complement activation and humoral immune response suggested that protein-protein interactions and immunoregulatory processes might play an important role in the development of LS in the Lundehund as it was previously proposed for PLE in the Soft Coated Wheaten Terrier [5].

Variants in the candidate gene *NOD1 (CARD4),* which could be found to harbor a Lundehund specific ROH region, have been shown to be highly susceptible to inflammatory bowel disease [30]. It was proposed that *NOD1* plays an important role in colonic epithelial protection against intracellular organisms similar to mucin genes. In Lundehund affected with atrophic gastritis an abnormal presence of mucous neck cells could be detected in the gastric mucosa [34]. We suppose that this gene, potentially under targeted selection in the Lundehund, might predispose this breed in general for gastrointestinal problems.

Further potential signatures of selection could be detected in the region of genes that might be involved in breed specific conformational traits like joint flexibility or polydactyly. According to Lundehund breed standard, the Lundehund was reported to have at least six toes at fore and hind limps [2, 18, 20]. Mapping analysis for hind limp specific canine preaxial polydactyly revealed a potentially causative mutation in *LMBR1* (DC-2) in western dog breeds [19]. The homozygous mutant DC-2 genotype was also found in the Lundehund [18] and could be confirmed in all 36 Lundehund in our study. Nevertheless, it was still unclear if this mutation could explain the characteristic phenotype in the Lundehund showing polydactyly at both fore and hind limps in contrast to the usually unchanged forelimb in dogs with polydactyly [19]. We found evidence in Lundehund consensus ROH regions for potential modifier genes that might play a role in limb development. *GREM2* was shown to be expressed in osteoblasts during in vivo skeletogenesis and involved in the regulation of bone formation genes [35, 36]. It was suggested that *GREM2* was bidirectionally regulated by bone morphogenetic protein 2 (BMP2) together with *GREM1*, a gene which was shown to be expressed along with *sonic hedgehog (SHH)* and play an important role in limb bud development [36, 37]. A similar effect was suggested for *PRRX1 (PRX1)*. Loss-of-function mutant *PRRX1/PRRX2* mice resulted in shortened zeugopods of the forelimbs and hindlimbs as well as postaxial polydactyly in the forelimb [38]. EHH analysis in the Lundehund revealed *PRRX2* in long-range homozygous haplotypes suggesting this gene as a candidate for Lundehund-polydactyly [17]. We assume that these genes under potential targeted selection could explain the specific phenotypic characteristic for a four-limb polydactyly in the Lundehund.

## Conclusions

In conclusion, our study of whole-genome sequencing data of Lundehund dogs suggests *LEPREL1*:g.139212C>G as a potential causative mutation for LS as well as *NOD1* as a potential precursor gene which might play a role in LS breed predisposition. In addition, we identified potential signatures of selection for characteristic breed specific traits whose targeted selection might have increased genetic risk factors for disease development.

The results of our analysis represent a significant step to identify the genetic background of Lundehund specific traits which still remain to be further investigated for proper understanding of the underlying complex genetic mechanisms.

## Methods

### Animals

Genomic DNA was obtained from blood samples of 36 Lundehund and 186 dogs of different dog breeds including Norwegian Buhund and Norrbottenspets using a standard saline precipitation method [39]. Lundehund specific phenotypes and affection-status were obtained on basis a questionnaire filled in by dog owners and veterinarians. Information about the pedigree, date of birth, date of euthanasia and signs of LS like diarrhea, vomiting and cachexia was recorded using this form. Further blood parameters were added from clinically examined dogs.

### Association analysis

In total 25 Lundehund (17 LS-affected and 8 LS-unaffected) were genotyped on the canine Illumina high density bead chip (Illumina) according to manufacturer’s protocols. Quality of genotyping data was controlled using a minor allele frequency (MAF) >0.05 and a genotyping rate per SNP >0.90. Filtering resulted in 93,882 SNPs with a mean genotyping rate of 99 %. Genome-wide association analysis was run using TASSEL version 3.0 [40]. Generalized linear model (GLM) with sex, inbreeding coefficients and three PCA’s was run to test the model. Quantile-quantile (Q-Q) plots for observed *versus* expected -log_10_P-values were calculated to control for population stratification using SAS/Genetics.

### Pedigree analysis

Pedigree analysis was performed using regressive logistic models and the procedure SEGREG of S.A.G.E. (Statistical Analysis for Genetic Epidemiology, Release 6.3: http://darwin.cwru.edu, 2012) to test the most likely mode of inheritance for LS. We tested the hypothesis for the μ model without any genetic component, monogenic inheritance, polygenic inheritance and mixed major gene inheritance with a polygenic component and an independently segregating major gene locus with two alleles.

### Whole-genome sequencing

Whole genome sequencing of Lundehund samples was performed on the Illumina MiSeq (Illumina, San Diego, CA) in paired-end mode. Libraries were prepared for one DNA-pool of three LS-affected Lundehund and for one DNA-pool of three LS-unaffected Lundehund using the Illumina Nextera DNA Sample Prep Kit according to standard protocols. We performed four runs for each pool with v2 Reagent Kits (Illumina) on a single lane flow cell (2 × 250 bp reads). Fastq-files were quality controlled using fastqc 0.11.3 [41]. Mapping to the reference genome CanFam 3.1.78 was performed using BWA 0.7.12 [42] followed by conversion into binary format using SAMtools 1.2 [43] and PCR duplicate marking using Picard tools (http://broadinstitute.github.io/picard/, version 1.130). Furthermore, we locally realigned reads and performed quality score recalibration and SNP calling using GATK [44]. In addition to the Lundehund fastq files we added five whole-genome sequences of one Korean Jindo Dog (DRR001566), one Afghan Hound (SRR1061643), two German Shepherd dogs (SRR1130247 and SRR1124304) and one Border Collie (SRR654728) from Sequence Read Archive (NCBI) to analysis. Quality of the data was controlled filtering variants for a read depth >2 and <1000 and quality values >20 (qual). Variant effects were predicted using the genetic variant annotation and effect prediction toolbox SNPEff version 4.1 b (2015-02-13) [45].

### ROH detection

ROHs were detected using a stringently quality-controlled autosomal dataset of 8,585,517 SNPs showing a minimum read depth of 3, a maximum read depth of 60, a minimum mean read depth of 12 and minor allele frequency 0.01 for all samples. Sliding windows of 500 SNPs were chosen for ROH analysis. Homozygous regions of >150 kb were detected as ROHs using PLINK, version 1.07 (http://pngu.mgh.harvard.edu/purcell/plink/). Based on the genome size covered with SNPs divided by the number of SNPs we estimated the minimum distance of SNPs as 0.3. A maximum of three SNPs with missing genotypes and three heterozygous SNPs were admitted in each window. ROHs were filtered for LS-specific and Lundehund specific ROH regions based on hom.summary data outputs from ROH analysis for each dog using SAS/Genetics, version 9.4 (Statistical Analysis System, Cary, NC). The homozygosity information of each SNP (1 = homozygosity, 0 = no homozygosity) was detected and compared to the other individuals in order to identify overlapping and private regions, the number of SNPs and length of the regions. These specific ROH regions were searched for variants predicted to have high or moderate effects (SNPEff).

### Functional annotation

Chromosomal positions of ROH regions were merged with Ensembl gene predictions (ensGene) using Galaxy intersection tool (https://usegalaxy.org/) [46, 47]. The obtained gene lists were converted into human orthologous genes using g:Profiler [48, 49] and tested for statistical overrepresentation in biological processes (PANTHER Overrepresentation Test; release 20150430, version 10.0) [50].

### Filtering analysis

Analysis of sequencing data was performed identifying variants with SNPEff-predicted high or moderate effects with a homozygous mutant genotype in the LS-affected pool, a heterozygous or homozygous wild-type genotype in LS-unaffected pool and a homozygous wild-type genotype in the other five dogs of different breeds using SAS/Genetics 9.4. Variants flagged with warnings about possible annotation accuracy problems were omitted from analysis. The candidate SNP in *LEPREL1* and further 23 SNPs were investigated for functional effects using the Variant Effect Predictor [51] for SIFT [21] predictions and PolyPhen [22]. Regions of SNPs that were predicted to be deleterious (SIFT) and possibly or probably damaging (PolyPhen) had been further investigated for a high density of mutations around the candidate SNP that could reference to annotation problems.

### Genotyping

Genotyping of the candidate SNPs *LEPREL1*:g.139212C>G, *KRT3*:g.2584C>T as well as the polydactyly-associated polymorphism in *LMBR1* (DC-2) was performed using restriction fragment length polymorphisms according to standard protocols [52]. DC-2 primers were obtained from previous study [18]. In addition the missense variants *CEP164*:g.57380G>T and *COL28A1*:g.159951T>A were validated using Kompetitive Allele Specific PCR (KASP) genotyping assays (LGC, Middlesex, UK) [53]. After the KASP standard thermal cycling touchdown protocol was run on a thermocycler TProfessional 96 (Biometra, Göttingen, Germany) using an annealing temperature of 61 °C (Additional file 12), allelic discrimination was performed on the ABI7300 sequence detection system (Applied Biosystems, Waltham, Massachusetts, USA).

### Sequencing of candidate genes

Sanger sequencing of the cDNA of *MB21D2* and *LEPREL1* was performed in one LS-affected Lundehund and one Great Dane as control dog. RNA was obtained from hair roots in stabilized RNALater reagent (Quiagen, Maryland, USA), transcribed into cDNA and used for PCR amplification according to a standardized protocol [54] (Additional file 13). Alignment and variant detection was performed using the analysis software Sequencher 4.8 (Genes Codes, Ann Arbor, MI, USA). Despite the use of Q-solution (Quiagen) as enhancer reagent and Gradient PCR for optimizing reaction conditions, the coding region of *FGF12* could not be amplified from these hair root samples at all.

## Abbreviations

CARD9, Caspase Recruitment Domain Family, Member 9; CCDC50, Coiled-Coil Domain Containing 50 ; cDNA, complementary DNA; CEP164, centrosomal protein 164 kDa; COL28A1, collagen, type XXVII, alpha 1; EHH, extended haplotype homozygosity; FGF12, fibroblast growth factor 12; GLM, generalized linear model; GREM2, gremlin 2, DAN family BMP antagonist; GWAS, genome-wide association analysis; KRT3, keratin 3; LEPREL1, leprecan-like 1; LMBR1, limb development membrane protein 1; LS, Lundehund syndrome; MB21D2, mab-21 domain containing 2; NOD1, Nucleotide-Binding Oligomerization Domain Containing 1; PLE, protein-losing enteropathy; PRRX1, paired related homeobox 1; ROH, runs of homozygosity; SHH, sonic hedgehog; SNV, single nucleotide variant

## Additional files

Additional file 1: Clinical data of 21 Lundehund with signs of LS. Blood parameters of total protein (TP), albumin (ALB), globulin (GLOB), fructosamine (F), alkaline phosphatase (ALKP), alanine aminotransferase (ALT), vitamin B12 (B12) and folic acid (FA) are shown. (DOCX 18 kb) Additional file 2: Pedigree of LS-affected Lundehund. In total, samples of twenty-one LS-affected and fifteen LS-unaffected Lundehund were available for analysis. A common pedigree for all these dogs shows a close relationship in-between all Lundehund dogs. Whole genome sequencing was performed in three affected and three LS-unaffected dogs (numbers in boxes) whereas genotyping on the Illumina high density bead chip was done for seventeen LS-affected and eight LS-unaffected Lundehund (numbers S1-S25). (TIF 2248 kb) Additional file 3: Results of complex segregation analysis using regressive logistic models for LS in the Lundehund. (DOCX 12 kb) Additional file 4: Runs of homozygosity for LS-affected Lundehund in 500-SNP windows. The chromosomal position of ROH regions, number of SNPs in these regions (n), size in base pairs (size_bp), canine genes (gene) and human orthologues (human gene) are shown. (XLSX 319 kb) Additional file 5: Private runs of homozygosity for LS-affected Lundehund in 500-SNP windows. The chromosomal position of ROH regions, number of SNPs in these regions (n), size in base pairs (size_bp), canine genes (gene) and human orthologues (human gene) are shown. (XLSX 60 kb) Additional file 6: Private runs of homozygosity for LS-unaffected Lundehund in 500-SNP windows. The chromosomal position of ROH regions, number of SNPs in these regions (n), size in base pairs (size_bp), canine genes (gene) and human orthologues (human gene) are shown. (XLSX 65 kb) Additional file 7: Runs of homozygosity for Lundehund specific regions in 500-SNP windows. The chromosomal position of ROH regions, number of SNPs in these regions (n), size in base pairs (size_bp), canine genes (gene) and human orthologues (human gene) are shown. (XLSX 88 kb) Additional file 8: Genotyping results for DC-2 mutation. The intronic LMBR1 SNP which is known to be associated with polydactyly (Park, 2008) was genotyped for all Lundehund and further 13 different dog breeds. (DOCX 13 kb) Additional file 9: Functional annotations for runs of homozygosity (ROH) in the Lundehund. PANTHER gene list analysis shows the proportion of gene hits against total number of process hits for genes detected in Lundehund consensus as well as LS-specific and private ROHs for LS-affected and LS-unaffected dogs. Detected ROHs were investigated for genes and their involvement in specific biologic processes. (DOCX 16 kb) Additional file 10: Allele frequencies of candidate SNPs. In total 186 samples of 17 breeds were genotyped for the LS-candidate SNPs *LEPREL1*:g.139212C>G, *KRT3*:g.2584C>T, *COL28A1*:g.159951T>A and *CEP164*:g.57380G>T. In *KRT3*:g.2584C>T and *CEP164*:g.57380G>T the mutant allele could be detected in other breeds whereas *LEPREL1*:g.139212C>G and *COL28A1*:g.159951T>A could not be found in any other dog breed than the Lundehund. (DOCX 14 kb) Additional file 11: Mutations detected in sequencing analysis of *MB21D2* and *LEPREL1*. None of the synonymous as well as 3 prime UTR mutations could be shown to be exclusively found in the Lundehund except *LEPREL1*:c.1849C>G. (DOCX 13 kb) Additional file 12: Primer sequences used for genotyping of candidate SNPs. The two variants located in KRT3 and LEPREL1 were genotyped by the use of restriction fragment length polymorphisms (RFLP). KASP-primers were used for genotyping CEP164 and COL28A1 variants. Primer pairs, amplicon size (AS) in base pairs (bp), annealing (AT), restriction enzyme and incubation temperature (IT) are shown. The polymorphism DC-2 in LMBR1 was detected using primer pairs previously published (Kropatsch, 2015). (DOCX 14 kb) Additional file 13: Sequences of primers used for the investigation of *MB21D2* and *LEPREL1* complementary DNA. The gene region, product sizes in base pairs (bp) and annealing temperatures are shown. (DOCX 13 kb)
