# Supplementary material for: Variant detection and runs of homozygosity in next generation sequencing data elucidate the genetic background of Lundehund syndrome
Source: BMC Genomics. 2016 Aug 2;17:535. doi: 10.1186/s12864-016-2844-6 (PMC4971756; doi:10.1186/s12864-016-2844-6)
Supplement: Additional file 1: — Clinical data of 21 Lundehund with signs of LS. Blood parameters of total protein (TP), albumin (ALB), globulin (GLOB), fructosamine (F), alkaline phosphatase (ALKP), alanine aminotransferase (ALT), vitamin B12 (B12) and folic acid (FA) are shown. (DOCX 18 kb) [file 12864_2016_2844_MOESM1_ESM.docx]

Additional file 1. Clinical data of 21 Lundehund with signs of LS. Blood parameters of total protein (TP), albumin (ALB), globulin (GLOB), fructosamine (F), alkaline phosphatase (ALKP), alanine aminotransferase (ALT), vitamin B12 (B12) and folic acid (FA) are shown.

| Dog | Sex | Age of the dog when first symptoms appeared  (months) | Died (years) | TP (g/dl) reference: 5.5-7.3 | ALB (g/dl) reference:3.1-4.7 | GLOB (g/dl) reference: 1.6-4.1 | F (umol/l) reference: 203- 377 | CA (mmol/l) reference:2.1-2.9 | ALT (U/L) reference: 10-100 | B12  (pg/ml) reference: 300-800 | FA (ng/ml) reference: 3.0-10.0 |
| --- | --- | --- | --- | --- | --- | --- | --- | --- | --- | --- | --- |
| Dog 1 | m | 48 | - | 2.79 | 1.17 | 1.62 | - | - | - | 200 | 10.2 |
| Dog 2 | m | 125 | 11.1 | 2.8 | 2.0 | 0.8 | - | - | 211 | - | - |
| Dog 3 | f | 120 | - | 5.7 | 2.7 | 3.0 | 187 | 2.4 | 101.2 | - | - |
| Dog 4 | m | NA | 14.2 | - | - | - | - | - | - | - | - |
| Dog 5 | f | NA | 5 | 5.0 | 1.9 | 3.1 | - | - | - | - | - |
| Dog 6 | m | NA | 8 | 4.1 | 2.3 | 1.8 | - | - | - | - | - |
| Dog 7 | f | 53 | 12.7 | 3.8 | 1.8 | 2.0 | 131 | 2.8 | 123 | - | - |
| Dog 8 | f | 29 | - | - | - | - | - | - | - | - | - |
| Dog 9 | f | NA | - | - | - | - | - | - | - | - | - |
| Dog 10 | m | 44 | 6.9 | 3.1 | 1.0 | 2.1 | 101 | 1.61 | 92.4 | - | - |
| Dog 11 | m | 48 | 4.0 | 4.1 | 1.7 | 2.31 | 154 | 1.65 | 901.6 | 133 | 14.28 |
| Dog 12 | m | 103 | - | - | - | - | - | - | - | - | - |
| Dog 13 | m | NA | 2.4 | - | - | - | - | - | - | - | - |
| Dog 14 | f | NA | - | 3.7 | - | - | - | - | - | - | - |
| Dog 15 | f | NA | - | 3.9 | 2.0 | 1.9 | - | - | - | - | - |
| Dog 16 | f | NA | 8.5 | 3.3 | - | - | - | - | - | - | - |
| Dog 17 | f | NA | 6.4 | 4.9 | 1.7 | 3.2 | - | - | - | - | - |
| Dog 18 | f | NA | 6.3 | - | - | - | - | - | - | - | - |
| Dog 19 | m | 96 | - | 6.2 | 2.7 | 3.5 | 202 | 2.3 | 108 | 565 | 13.4 |
| Dog 20 | m | 34 | - | 5.9 | 2.7 | 3.2 | 206 | 2.4 | 93 | 506 | 11.0 |
| Dog 21 | f | NA | - | - | - | - | - | - | - | - | - |
