# Supplementary material for: Variant detection and runs of homozygosity in next generation sequencing data elucidate the genetic background of Lundehund syndrome
Source: BMC Genomics. 2016 Aug 2;17:535. doi: 10.1186/s12864-016-2844-6 (PMC4971756; doi:10.1186/s12864-016-2844-6)
Supplement: Additional file 3: — Results of complex segregation analysis using regressive logistic models for LS in the Lundehund. (DOCX 12 kb) [file 12864_2016_2844_MOESM3_ESM.docx]

Additional file 3. Results of complex segregation analysis using regressive logistic models for LS in the Lundehund.

| Hypothesis | -2InL | AIC |
| --- | --- | --- |
| Saturated model | 52.53 | 68.53 |
| µ model | 57.93 | 61.93 |
| Recessive gene effect | 57.93 | 63.93 |
| Dominant gene effect | 56.64 | 64.65 |
| Arbitrary gene effect | 54.20 | 66.20 |
| Polygenic | 54.27 | 62.27 |
| Recessive major gene effect | 52.68 | 64.68 |
| Dominant major gene effect | 54.26 | 68.26 |
| Arbitrary major gene effect | 54.26 | 68.26 |
