# Supplementary material for: Variant detection and runs of homozygosity in next generation sequencing data elucidate the genetic background of Lundehund syndrome
Source: BMC Genomics. 2016 Aug 2;17:535. doi: 10.1186/s12864-016-2844-6 (PMC4971756; doi:10.1186/s12864-016-2844-6)
Supplement: Additional file 8: — Genotyping results for DC-2 mutation. The intronic LMBR1 SNP which is known to be associated with polydactyly (Park, 2008) was genotyped for all Lundehund and further 13 different dog breeds. (DOCX 13 kb) [file 12864_2016_2844_MOESM8_ESM.docx]

Additional file 8. Genotyping results for DC-2 mutation. The intronic *LMBR1* SNP which is known to be associated with polydactyly (Park, 2008) was genotyped for all Lundehund and further 13 different dog breeds.

| DC-2 | Number of genotyped dogs (n) | G/G  (wild type) | G/A | A/A |
| --- | --- | --- | --- | --- |
| Lundehund | 36 | 0 | 0 | 36 |
| Norrbottenspets | 3 | 3 | 0 | 0 |
| Norwegian Buhund | 3 | 0 | 0 | 3 |
| Russian European Laika | 1 | 1 | 0 | 0 |
| Bernese Mountain Dog | 6 | 0 | 4 | 2 |
| German Shepherd | 6 | 5 | 1 | 0 |
| Akita | 1 | 1 | 0 | 0 |
| Dalmatian | 2 | 2 | 0 | 0 |
| Afghan Hound | 1 | 1 | 0 | 0 |
| Korean Jindo Dog | 1 | 1 | 0 | 0 |
| Border Collie | 1 | 1 | 0 | 0 |
| Briard | 5 | 0 | 0 | 5 |
| Irish Wolfhound | 4 | 3 | 0 | 1 |
| Tibetan Terrier | 2 | 1 | 1 | 0 |
| total | 72 | 19 | 6 | 47 |
