# Supplementary material for: Variant detection and runs of homozygosity in next generation sequencing data elucidate the genetic background of Lundehund syndrome
Source: BMC Genomics. 2016 Aug 2;17:535. doi: 10.1186/s12864-016-2844-6 (PMC4971756; doi:10.1186/s12864-016-2844-6)
Supplement: Additional file 9: — Functional annotations for runs of homozygosity (ROH) in the Lundehund. PANTHER gene list analysis shows the proportion of gene hits against total number of process hits for genes detected in Lundehund consensus as well as LS-specific and private ROHs for LS-affected and LS-unaffected dogs. Detected ROHs were investigated for genes and their involvement in specific biologic processes. (DOCX 16 kb) [file 12864_2016_2844_MOESM9_ESM.docx]

Additional file 9. Functional annotations for runs of homozygosity (ROH) in the Lundehund. PANTHER gene list analysis shows the proportion of gene hits against total number of process hits for genes detected in Lundehund consensus as well as LS-specific and private ROHs for LS-affected and LS-unaffected dogs. Detected ROHs were investigated for genes and their involvement in specific biologic processes.

| PANTHER gene ontology terms | ROH regions in LS-affected dogs (%) | Private ROH regions for LS-affected dogs (%) | Private ROH regions for LS-unaffected dogs (%) | Lundehund consensus ROH regions (%) |
| --- | --- | --- | --- | --- |
| cellular component organization or biogenesis (GO:0071840) | 4.3 | 4.7 | 4.2 | 4.7 |
| cellular process (GO:0009987) | 21.1 | 19.5 | 20.9 | 20.5 |
| localization (GO:0051179) | 8.4 | 9.3 | 9.0 | 8.3 |
| apoptotic process (GO:0006915) | 1.6 | 1.3 | 1.4 | 1.5 |
| reproduction (GO:0000003) | 1.0 | 1.4 | 1.5 | 1.1 |
| biological regulation (GO:0065007) | 11.1 | 11.4 | 12.3 | 10.2 |
| response to stimulus (GO:0050896) | 6.9 | 6.5 | 5.9 | 7.1 |

Additional file 9 continued.

| PANTHER gene ontology terms | ROH regions in LS-affected dogs (%) | Private ROH regions for LS-affected dogs (%) | Private ROH regions for LS-unaffected dogs (%) | Lundehund consensus ROH regions (%) |
| --- | --- | --- | --- | --- |
| developmental process (GO:0032502) | 7.7 | 7.6 | 6.4 | 7.9 |
| rhythmic process (GO:0048511) | 0.0 | 0.0 | 0.0 | 0.1 |
| multicellular organismal process (GO:0032501) | 5.4 | 5.6 | 6.0 | 5.0 |
| locomotion (GO:0040011) | 0.2 | 0.2 | 0.2 | 0.5 |
| biological adhesion (GO:0022610) | 2.0 | 1.5 | 1.7 | 2.2 |
| metabolic process (GO:0008152) | 25.4 | 26.7 | 27.3 | 25.3 |
| immune system process (GO:0002376) | 4.7 | 4.3 | 3.2 | 5.7 |
| cell killing (GO:0001906) | 0.1 | 0.0 | 0.0 | 0.0 |
