# Supplementary material for: Variant detection and runs of homozygosity in next generation sequencing data elucidate the genetic background of Lundehund syndrome
Source: BMC Genomics. 2016 Aug 2;17:535. doi: 10.1186/s12864-016-2844-6 (PMC4971756; doi:10.1186/s12864-016-2844-6)
Supplement: Additional file 10: — Allele frequencies of candidate SNPs. In total 186 samples of 17 breeds were genotyped for the LS-candidate SNPs LEPREL1:g.139212C>G, KRT3:g.2584C>T, COL28A1:g.159951T>A and CEP164:g.57380G>T. In KRT3:g.2584C>T and CEP164:g.57380G>T the mutant allele could be detected in other breeds whereas LEPREL1:g.139212C>G and COL28A1:g.159951T>A could not be found in any other dog breed than the Lundehund. (DOCX 14 kb) [file 12864_2016_2844_MOESM10_ESM.docx]

Additional file 10. Allele frequencies of candidate SNPs. In total 186 samples of 17 breeds were genotyped for the LS-candidate SNPs *LEPREL1*:g.139212C>G, *KRT3*:g.2584C>T, *COL28A1*:g.159951T>A and *CEP164*:g.57380G>T. In *KRT3*:g.2584C>T and *CEP164*:g.57380G>T the mutant allele could be detected in other breeds whereas *LEPREL1*:g.139212C>G and *COL28A1*:g.159951T>A could not be found in any other dog breed than the Lundehund.

| Breed | Number of samples | Minor allele (G) frequency  LEPREL1:g.139212C>G | Minor allele (T) frequency  KRT3:g.2584C>T | Minor allele (T) frequency  COL28A1:g.159951T>A | Minor allele (T) frequency  CEP164:g.57380G>T |
| --- | --- | --- | --- | --- | --- |
| Appenzeller Mountain Dog | 16 | 0.00 | 0.00 | 0.00 | 0.00 |
| Bernese Mountain Dog | 16 | 0.00 | 0.00 | 0.00 | 0.47 |
| Wire-haired Dachshound | 16 | 0.00 | 0.00 | 0.00 | 0.00 |
| German Wirehaired Pointer | 16 | 0.00 | 0.09 | 0.00 | 0.00 |
| Great Dane | 16 | 0.00 | 0.00 | 0.00 | 0.16 |
| German Pinscher | 8 | 0.00 | 0.00 | 0.00 | 0.06 |
| German Shepherd | 16 | 0.00 | 0.00 | 0.00 | 0.00 |
| Doberman Pinscher | 16 | 0.00 | 0.00 | 0.00 | 0.06 |
| Entlebucher Mountain Dog | 16 | 0.00 | 0.00 | 0.00 | 0.00 |
| Greater Swiss Mountain Dog | 15 | 0.00 | 0.00 | 0.00 | 0.00 |
| Hovawart | 1 | 0.00 | 0.00 | 0.00 | 0.00 |
| Norrbottenspets | 3 | 0.00 | 0.40 | 0.00 | 0.00 |
| Norwegian Buhund | 3 | 0.00 | 0.00 | 0.00 | 0.00 |
| Russian European Laika | 1 | 0.00 | 0.00 | 0.00 | 0.00 |
| Shar-Pei | 16 | 0.00 | 0.00 | 0.00 | 0.00 |
| Tibetan Terrier | 10 | 0.00 | 0.00 | 0.00 | 0.00 |
| Shetland Sheepdog | 1 | 0.00 | 0.00 | 0.00 | 0.00 |
| Total | 186 | 0.00 | 0.49 | 0.00 | 0.75 |
