# Supplementary material for: Variant detection and runs of homozygosity in next generation sequencing data elucidate the genetic background of Lundehund syndrome
Source: BMC Genomics. 2016 Aug 2;17:535. doi: 10.1186/s12864-016-2844-6 (PMC4971756; doi:10.1186/s12864-016-2844-6)
Supplement: Additional file 11: — Mutations detected in sequencing analysis of MB21D2 and LEPREL1. None of the synonymous as well as 3 prime UTR mutations could be shown to be exclusively found in the Lundehund except LEPREL1:c.1849C>G. (DOCX 13 kb) [file 12864_2016_2844_MOESM11_ESM.docx]

Additional file 11. Mutations detected in sequencing analysis of *MB21D2* and *LEPREL1*. None of the synonymous as well as 3 prime UTR mutations could be shown to be exclusively found in the Lundehund except *LEPREL1*:c.1849C>G.

| ECA | Gene | Polymorphism name | Type | Base change | Source | Genotype Lundehund | Genotype reference dog |
| --- | --- | --- | --- | --- | --- | --- | --- |
| 34 | *MB21D2* | MB21D2:c.156G>T | synonymous | - | exon 2 | T/T | G/T |
| 34 | *MB21D2* | MB21D2:c.444C>T | synonymous | - | exon 2 | T/T | C/T |
| 34 | *MB21D2* | MB21D2:c.2467A>C  (rs23859516) | - | - | 3’UTR | C/C | C/C |
| 34 | *LEPREL1* | *LEPREL1*:c.1209A>G | synonymous | - | exon 7 | G/G | G/G |
| 34 | *LEPREL1* | *LEPREL1*:c.1224G>A | synonymous | - | exon 7 | A/A | A/A |
| 34 | *LEPREL1* | *LEPREL1*:c.1473A>G | synonymous | - | exon 10 | A/A | A/G |
| 34 | *LEPREL1* | *LEPREL1*:c.1849C>G  *(LEPREL1*:g.139212C>G) | non-synonymous | E>Q | exon 13 | G/G | C/C |
