# Supplementary material for: Variant detection and runs of homozygosity in next generation sequencing data elucidate the genetic background of Lundehund syndrome
Source: BMC Genomics. 2016 Aug 2;17:535. doi: 10.1186/s12864-016-2844-6 (PMC4971756; doi:10.1186/s12864-016-2844-6)
Supplement: Additional file 12: — Primer sequences used for genotyping of candidate SNPs. The two variants located in KRT3 and LEPREL1 were genotyped by the use of restriction fragment length polymorphisms (RFLP). KASP-primers were used for genotyping CEP164 and COL28A1 variants. Primer pairs, amplicon size (AS) in base pairs (bp), annealing (AT), restriction enzyme and incubation temperature (IT) are shown. The polymorphism DC-2 in LMBR1 was detected using primer pairs previously published (Kropatsch, 2015). (DOCX 14 kb) [file 12864_2016_2844_MOESM12_ESM.docx]

Additional file 12. Primer sequences used for genotyping of candidate SNPs. The two variants located in *KRT3* and *LEPREL1* were genotyped by the use of restriction fragment length polymorphisms (RFLP). KASP-primers were used for genotyping *CEP164* and *COL28A1* variants. Primer pairs, amplicon size (AS) in base pairs (bp), annealing (AT), restriction enzyme and incubation temperature (IT) are shown. The polymorphism DC-2 in *LMBR1* was detected using primer pairs previously published (Kropatsch, 2015).

| CFA | Gene | Polymorphism | Forward primer(s) (5’-3’) | Reverse primer (5’-3’) | AS (bp) | AT (°C) | Restriction enzyme | IT  (°C) |
| --- | --- | --- | --- | --- | --- | --- | --- | --- |
| 27 | *KRT3* | KRT3:g.2584C>T | GGATGGTAAGGAGGATGTGTTC | GAGGACTGACTGGTTCTTGTCC | 423 | 60 | HpyCH4IV | 37 |
| 34 | *LEPREL1* | LEPREL1:g.139212C>G | CTGGTCAGTTTGTCACGTCTTC | GCAAGATTGAGATGAAGCAATG | 216 | 60 | BsaJI | 60 |
| 5 | *CEP164* | CEP164:g.57380G>T | GGCTCCTGACCTCCTCCAG-FAM  CAGGCTCCTGACCTCCTCCAT-VIC | AGGCCCTGGAAGATGTGCGCAA | 45 | 61 (26 cycles) | - | - |
| 14 | *COL28A1* | COL28A1:g.159951T>A | GTTGAATCTATTTCCTGAGCCATTACA-FAM GTTGAATCTATTTCCTGAGCCATTACT-VIC | ACTCCTGCGCCCGCTTTTGGTT | 56 | 61 (35 cycles) | - | - |
| 16 | *LMBR1* | LMBR1:g.19380592G>A (DC-2) | GCAAATGTATCACAGACATTGAC | GATTGAGAAATAAGATCAATTTGATAAACA | 749 | 59 | Taqα1 | 65 |
