# Supplementary material for: Variant detection and runs of homozygosity in next generation sequencing data elucidate the genetic background of Lundehund syndrome
Source: BMC Genomics. 2016 Aug 2;17:535. doi: 10.1186/s12864-016-2844-6 (PMC4971756; doi:10.1186/s12864-016-2844-6)
Supplement: Additional file 13: — Sequences of primers used for the investigation of MB21D2 and LEPREL1 complementary DNA. The gene region, product sizes in base pairs (bp) and annealing temperatures are shown. (DOCX 13 kb) [file 12864_2016_2844_MOESM13_ESM.docx]

Additional file 13. Sequences of primers used for the investigation of *MB21D2* and *LEPREL1* complementary DNA*.* The gene region, product sizes in base pairs (bp) and annealing temperatures are shown.

| Gene | Gene region | Forward primer (5’-3’) | Reverse primer (5’-3’) | Product size (bp) | Annealing temperature (°C) |
| --- | --- | --- | --- | --- | --- |
| *MB21D2* | exon 1-2 | ATTATTCGCCAGCACGACCA | CCTGCATGAGGCTACTAGAGA | 802 | 58 |
| *MB21D2* | exon 2 | GGGAATACGACGACCAGATAG | CCTGCATGAGGCTACTAGAGA | 780 | 58 |
| *MB21D2* | exon 2 | TCATCAGTGGGTTTTACTTGG | TCAGCATCACAAACCACACTA | 759 | 58 |
| *MB21D2* | exon 2 | GTTTTTCTGAGCATCTTGCTG | TCAGCATCACAAACCACACTA | 92 | 58 |
| *MB21D2* | exon 2 | GTTTTTCTGAGCATCTTGCTG | ACATTGGAACTCTGACAATGG | 807 | 58 |
| *MB21D2* | exon 2 | TCCAGCCTAGATTGGATCTG | AGCATTTCAACAGTGGATGTC | 759 | 58 |
| *MB21D2* | exon 2 | AGAACTTTGGATGGTTTGGAG | CAACCTGTGAGTCTGGACATT | 819 | 58 |
| *MB21D2* | exon 2 | AAATCACTTGCCAGGTTTAGG | GGTGGCTCATGTTTTTCTTTC | 823 | 58 |
| *LEPREL1* | exon10-3’UTR | GCAACTGTCCTGAAAGCACTC | AGCATCCTCAGGAAGAGAAGG | 745 | 60 |
| *LEPREL1* | exon1-6 | CGCAGAGTGCCCTACAACTAC | TATGACGCTTCACGAACATTG | 688 | 58 |
| *LEPREL1* | exon3-9 | GAGGCTGATGACTTTGAGCTG | TGACAGGACGTTATCCAGGAG | 753 | 60 |
| *LEPREL1* | exon7-14 | ATTGGATCCGATATGGAGGAC | AAACTGATCATACGCCCACAC | 739 | 64 |
| *LEPREL1* | exon8-14 | AGGGTGGCCCTCTACTCTATG | AGTGGGTCCAAGGTAAACCAC | 700 | 60 |
